# Inhibition of Oxidation of Aqueous Emulsions of Omega-3 Fatty Acids and Fish Oil by Phloretin and Phloridzin

**DOI:** 10.3390/molecules15010251

**Published:** 2010-01-11

**Authors:** H. P. Vasantha Rupasinghe, Afsana Yasmin

**Affiliations:** Tree Fruit Bio-product Research Program, Nova Scotia Agricultural College, P.O. Box 550, Truro, NS, B2N 5E3, Canada

**Keywords:** phloretin, phloridzin, dihydrochalcone, lipid oxidation, peroxyl radical, antioxidant

## Abstract

The antioxidant properties of two apple dihydrochalcones, namely phloretin and phloridzin, were evaluated and compared with those of α-tocopherol and butylated hydroxytoluene (BHT). The effects were studied in an oil-in-water emulsion system containing methyl linolenate (ML), methyl eicosapentaenoate (MEPA), and methyl docosahexaenoate (MDHA) in which oxidation was initiated by the peroxyl radical generator 2,2-azobis(2-amidinopropane) dihydrochloride (AAPH) and in fish oil where oxidation was initiated thermally. In the emulsion system, phloretin (1 and 5 mM) completely inhibited the oxidation of ML tested as evidenced by the thiobarbituric acid reactive substances (TBARS) assay. Under the same conditions, phloridzin was less effective than phloretin, but still more effective than α-tocopherol. Both phloretin and phloridzin molecules had a marginal inhibitory effect against oxidation of fish oil induced by heating at 70 °C for 3 hours, when compared to BHT. These results indicate that phloretin and phloridzin have the potential to suppress lipid oxidation in polyunsaturated fatty acid (PUFA) containing foods.

## 1. Introduction

Polyunasaturated fatty acids (PUFA) are highly oxidizable molecules and reactive with hydroxyl and peroxyl radicals due to the presence of multiple double bonds [1]. Oxidation of PUFA in foods produces hydroperoxides, aldehydes, and polymeric substances, resulting in adverse health effects and potentially leading to chronic diseases such as cancer, cardiovascular and neurological diseases [2,3,4]. Lipid oxidation is a major contributor to rancid off-flavors in foods, but it can be controlled by the use of antioxidants which readily stabilize free radicals by donating an electron or a hydrogen atom and thus prevent lipid peroxidation [5]. 

The importance of dietary PUFA such as linolenic acid (C18:3n-3), eicosapentaenoic acid (EPA, C20:5n-3), docosahexaenoic acid (DHA, C22:6n-3) as an essential fatty acid in fish oil has been reported [6,7]. Recently, the use of omega-3 PUFA as value-added food ingredients has increased [8]; hence, prevention of PUFA oxidation has received renewed attention. Synthetic antioxidants such as butylated hydroxytoluene (BHT) have been used to prevent oxidation of PUFA but their future use has been questioned due to their potential carcinogenic properties [9,10]. Therefore, the current attention has been concentrated on the discovery and use of natural antioxidants. 

**Figure 1:**
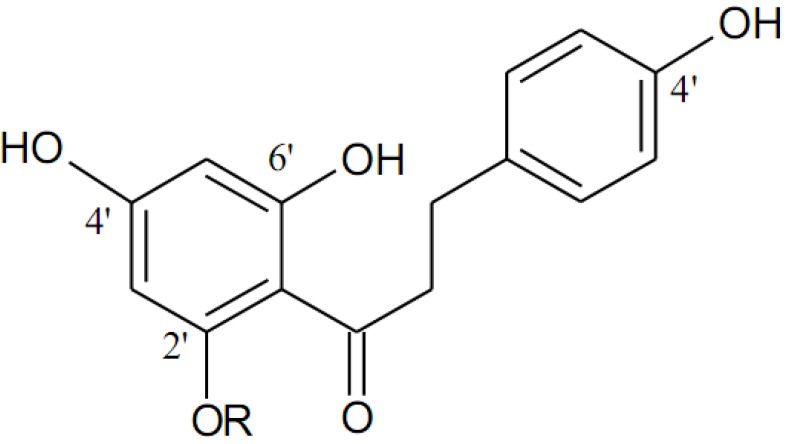
Chemical structures of phloretin (R=H) and phloridzin (R=D-glucose).

Fruits and vegetables and their processing by-products are one of the most important sources of natural antioxidants, due to the abundance of phenolic compounds such as flavonoids [11]. Phloretin (β-(4-hydroxyphenyl)-1-(2’,4’,6’-trihydroxypropiophenone) and its glucoside phloridzin (phloretin-2-β-D-glucose; also called phlorhizin or phlorizin) (Figure 1), which belong to the dihydrochalcone (bicylic flavonoid) flavonoid sub-class, are abundantly found in apples [12]. In addition to their free radical scavenging properties [13], dihydrochalcones possess numerous biological activities [14]. Phloretin and phloridzin have been identified as potent antioxidants in the inhibition of lipid peroxidation in isolated liver microsomes of rats [15]. The antioxidant protection of omega-3 PUFA and fish oil by apple skin extracts containing phloretin and phloridzin has been reported [16], but the antioxidant protection of omega-3 PUFA afforded by these two dihydrochalcones individually has not been reported, thus the objective of this research was to evaluate the antioxidant activity of phloretin and phloridzin in reducing the oxidation of some representative PUFA methyl esters: methyl linolenate (ML), methyl eicosapentaenate (MEPA) and methyl docosahexaenoate (MDHA) in an oil-in-water emulsion and fish oil in comparison with α-tocopherol and BHT. 

## 2. Results and Discussion

In order to investigate the antioxidant ability of two predominant dihydrochalcones of apples, phloretin and phloridzin, in protecting PUFA from oxidation, two model systems, namely peroxyl radical-induced oxidation of ML, MEPA and MDHA in oil-in-water emulsion and heat-induced oxidation of fish oil were employed. The efficacy of the two studied compounds was also compared with two commercial food antioxidants, α-tocopherol and BHT. The optimum induction period was the same for the studied fatty acids methyl esters in water-in-oil emulsion system and also the resultant secondary products of lipid oxidation as estimated by the thiobarbituric acid reactive substances (TBARS) assay. 

In general, the formation of TBARS was lower when either phloretin, phloridzin, α-tocopherol or BHT were added to the fatty acid methyl esters in comparison to the respective controls without antioxidants (Table 1). Interestingly, phloretin and phloridzin protected oxidation of fatty acid methyl esters in emulsion more efficiently than α-tocopherol (Table 1). Higher concentrations of phloretin (5 mM) exhibited 94% and 74% inhibition of peroxyl radical-induced oxidation of MEPA and MDHA, respectively. Among the tested compounds, BHT (0.1, 1, and 5 mM) protected completely against the oxidation of fatty acid methyl esters.

**Table 1:** The effect of phloretin and ploridzin on the peroxyl radical-induced oxidation in oil-in-water emulsion system of ML, MEPA and MDHA.

Antioxidant	Concentration (mmole per L)	% Inhibition		
		ML	MEPA	MDHA
Phloretin	0.1	72.5 ± 5.5^ c^	66.0 ± 5.5^ c^	53.4 ± 3.9^ c^
	1	100^ a^	90.6 ± 4.6^ a^	74.1 ± 4.4^ a^
	5	100^ a^	93.6 ± 4.0^ a^	76.9 ± 1.8^ a^
Phloridzin	0.1	53.4 ± 3.6 ^d^	49.1 ± 4.5 ^d^	40.1 ± 1.8^ d^
	1	81.8 ± 4.9^ b^	73.7 ± 5.4^ b^	61.7 ± 2.7^ b^
	5	100^ a^	89.3 ± 3.5^ a^	71.1 ± 2.5^ a^
α-Tocopherol	0.1	2.8 ± 1.9^ g^	2.3 ± 1.0^ g^	3.0 ± 2.1^ g^
	1	11.2 ± 2.7^ f^	9.8 ± 3.3^ f^	8.8 ± 1.7^ f^
	5	27.3 ± 2.7^ e^	24.7 ± 2.4^ e^	19.8 ± 2.2^ e^
BHT	0.1	100 ^a^	92.8 ± 4.6^ a^	74.0 ± 1.6^ a^
	1	100^ a^	92.9 ± 4.3^ a^	77.0 ± 1.0^ a^
	5	100^ a^	92.9 ± 4.0^ a^	79.4 ± 1.1^ a^

Data represent the mean (n = 6) of two individual experiments ± SD. Superscript in columns without letters in common differ significantly (*P* < 0.05).

In the fish oil system, allowed to oxidize at 70 °C for 3 hr, higher concentrations of phloretin, phloridzin, α-tocopherol and BHT were required to prevent the oxidation when compared to oil-in-water fatty acid methyl ester emulsions (Table 2). The inhibition was higher than 60% when 10 or 50 mM α-tocopherol or BHT were added to fish oil. In contrast, phloretin and phloridzin exhibited a low to marginal inhibition of fish oil oxidation and were less effective than α-tocopherol and BHT at the equivalent concentrations (Table 2).

**Table 2:** The effect of phloretin and phloridzin on the heat-induced oxidation of fish oil.

Antioxidant	Concentration (mmole per L)	% Inhibition of oxidation
Phloretin	1	14.3 ± 2.2^ j^
	10	23.3 ± 3.5^ ij^
	50	30.7 ± 2.2^ gh^
Phloridzin	1	24.5 ± 2.9 ^hi^
	10	33.9 ± 3.2^ fg^
	50	48.6 ± 2.8^ e^
α-Tocopherol	1	27.6 ± 6.9^ ghi^
	10	68.4 ± 4.3^ c^
	50	79.3 ± 2.3^ b^
BHT	1	40.3 ± 3.9^ f^
	10	60.5 ± 3.0^ d^
	50	90.2 ± 1.9^ a^

Data represent the mean (n = 6) of two independent experiments ± SD. Superscripts in columns without letters in common differ significantly (*P* < 0.05).

In the oil-in-water emulsion, the lipophilic phloretin exhibited greater antioxidant activity when compared to its relatively less lipophilic glucoside phloridzin. The effectiveness of antioxidants depends on different interfacial affinities in different lipid systems, as explained by the so-called ‘polar paradox’ theory that indicates lipophilic antioxidants are more effective in lipid emulsions [17]. Similarly, phloretin has been found to be a potent antioxidant compared to phloridzin in scavenging peroxynitrite [15], hydroxyl radical [18], and 1,1-diphenyl-2-picrylhydrazyl radical [14], as well as in inhibition of lipid peroxidation of isolated rat liver microsomes [15]. The antioxidant moiety of phloretin is the 2,6-dihydroxyacetophenone nucleus, which enhances the electron donating effects of phloretin and subsequently allows formation of a stable free radical [15]. In phloridzin, the hydroxyl groups next to the carbonyl group is substituted by a sugar moiety (Figure 1), thus hindering the formation of 2,6-dihydroxyacetophenone [15]. Furthermore, the presence of a hydroxyl group at the 2′-position of dihydrochalcone A ring is an essential pharmacophore for the radical scavenging potential of both phloretin and phloridzin [19]. 

## 3. Experimental

### 3.1. Chemicals

Methyl linolenate, MEPA, and MDHA were obtained from Nu-Chek (Elysian, MN, USA). The fish oil [03/55 TG fish oil, CFIA reg. 3529; 61% EPA, 4.3% DHA, 17.6 monounsaturated, 77.6 polyunsaturated fatty acid by weight of total fatty acids] was a generous gift from Ocean Nutrition Canada, Dartmouth, NS, Canada. Phloretin, phloridzin, α-tocopherol, BHT, thiobarbituric acid (TBA) and trichloroacetic acid (TCA) were purchased from Sigma-Aldrich (St. Louis, MO, USA). 2,2-Azobis(2-amidinopropane) dihydrochloride (AAPH) was acquired from Wako Chemicals (Richmond, VA, USA). Microplates (96-well) were purchased from Fisher Scientific (Ottawa, ON, Canada). All other chemicals were obtained from Fisher Scientific. 

### 3.2. Preparation of oil-in-water emulsion system

Phloretin, phloridzin, α-tocopherol and BHT were used at 0.1, 1.0 and 5.0 mM to inhibit peroxyl radical induced oxidation of ML, MEPA, and MDHA individually. The emulsions of each substrate were prepared following a previously described method [20]. Briefly, PUFA substrate (1.5 mg per mL) was suspended in a buffer/emulsifer (0.05 M TRIS-HCl, 0.15 M KCl and 1% Tween 20, pH 7) by homogenization for 20 s using a Polytron homogenizer (PCU Drehzahlregler, Switzerland). 

### 3.3. Induction of oxidation

For induction of oxidation of oil-in-water emulsion, freshly prepared peroxyl radical generator (0.1 M AAPH, 100 μL) was added to emulsion (0.9 mL) in disposable borosilicate glass tubes (13 × 100 mm) and incubated at 37 °C for 24 hours using a shaker oven at 150 rpm (model Apollo HP50, CLP Tools, San Diego, CA, USA). Fatty acid emulsions with no antioxidant were included as blanks for each experiment. For the fish oil (bulk oil) model system, 10, 50 or 100 mM of phloretin, phloridzin, α-tocopherol, or BHT (100 μL) were transferred into disposable borosilicate glass tubes (13 × 100 mm), evaporated to complete dryness under a stream of nitrogen and then fish oil (100 μL) was added, and the mixture vortexed, and sonicated for 2 min at 30 °C to facilitate the incorporation of tested compounds into the oil. The oxidation of fish oil containing antioxidants was induced by exposure to heat (70 °C) for 3 hours using a shaker oven at 150 rpm (model Apollo HP50, CLP Tools, San Diego, CA, USA). For each treatment, triplicate samples were subjected to oxidation and the experiment was repeated twice independently. 

### 3.4. Thiobarbituric acid reactive substances (TBARS) assay

After the completion of the oxidation treatment, TBARS were quantified as previously described [20]. One-hundred microliters of 2% BHT in ethanol were added to the test tubes to stop the oxidation process. The TBA reagent [1 mL of 15% (w/v) trichloroacetic acid and 0.375% (w/v) TBA in 0.25 M HCl] was then added and mixed. The reaction mixture was placed in an 80 °C water bath for 15 min, after which the samples and standards were cooled to room temperature and centrifuged at 2,000 rpm for 15 min (model Durafuge 300, Precision Scientific, Asheville, NC, USA). The absorbance of the supernatant was then measured at 532 nm using 96-well microplates in the FLUOstar OPTIMA plate reader (BMG Labtech, Durham, NC, USA). The outer wells of the microplates were not included. After the subtraction of blank values, absorbance values were used for calculation of % inhibition of oxidation:

% Inhibition of oxidation = [1-(sample absorbance/control absorbance)] × 100

### 3.5. Statistical analysis

All experiments were performed independently twice with three replicates per each independent experiment and the results presented as mean±standard deviation (n = 6). The design for all the parameters was randomized blocks design (RBD) with experimental run as the blocking factor, and compound and concentration as factors of interest. The assumptions of normality of error terms were tested using the Anderson-Darling test. Assumptions of constant variance were checked by plotting residual versus fits scatter diagram [21]. The data were analyzed using the general linear model (GLM) procedure of the SAS Institute, Inc. Significant differences among means were determined by the Tukey’s Studentized Range test at α = 0.05. 

## 4. Conclusions

In summary, the results of the present study suggest that phloretin and phloridzin, two plant secondary molecules found mainly in apples, have a significant effect in preventing peroxyl radical induced oxidation of PUFA in aqueous emulsions. Phloretin was more powerful antioxidant compared to its glucoside phloridzin in the emulsions. Further investigation of dihydrochalcones is necessary to explore the potential of these natural flavonoids as stabilizers in PUFA containing food and nutraceutical products.
